# β-Alanine Supplementation in Combat Sports: Evaluation of Sports Performance, Perception, and Anthropometric Parameters and Biochemical Markers—A Systematic Review of Clinical Trials

**DOI:** 10.3390/nu15173755

**Published:** 2023-08-28

**Authors:** Diego Fernández-Lázaro, Emma Marianne Fiandor, Juan F. García, Natalia Busto, Mirian Santamaría-Peláez, Eduardo Gutiérrez-Abejón, Enrique Roche, Juan Mielgo-Ayuso

**Affiliations:** 1 Department of Cellular Biology, Genetics, Histology and Pharmacology, Faculty of Health Sciences, University of Valladolid, Campus of Soria, 42004 Soria, Spain; 2 Neurobiology Research Group, Faculty of Medicine, University of Valladolid, 47005 Valladolid, Spain; 3 Research Group “Nutrition and Physical Activity”, Spanish Nutrition Society “SEÑ”, 28010 Madrid, Spain; eroche@umh.es (E.R.); jfmielgo@ubu.es (J.M.-A.); 4 Faculty of Physical Activity and Sport Sciences, European University, 28670 Villaviciosa de Odón, Spain; emma.ft14@gmail.com; 5 Department of Mechanical, Informatics and Aerospatial Engineering, University of Leon, 24071 Leon, Spain; 6 Department of Health Sciences, Faculty of Health Sciences, University of Burgos, 09001 Burgos, Spain; 7 Pharmacological Big Data Laboratory, Faculty of Medicine, University of Valladolid, 47005 Valladolid, Spain; 8 Pharmacy Directorate, Castilla y León Health Council, 47007 Valladolid, Spain; 9 Department of Applied Biology-Nutrition, Institute of Bioengineering, University Miguel Hernández, 03202 Elche, Spain; 10 Alicante Institute for Health and Biomedical Research (ISABIAL), 03010 Alicante, Spain; 11 CIBER Fisiopatología de la Obesidad y Nutrición (CIBEROBN), Instituto de Salud Carlos III (ISCIII), 28029 Madrid, Spain

**Keywords:** β-alanine, nutritional ergogenic aids, buffers, combat athletes, sports performance, perceptual parameters, anthropometric measures, biochemical markers

## Abstract

β-alanine does not have an ergogenic effect by itself, but it does as a precursor for the synthesis of carnosine in human skeletal muscle. β-alanine and carnosine together help improve the muscles’ functionality, especially in high-intensity exercises such as combat sports. Therefore, β-alanine could be considered a nutritional ergogenic aid to improve sports performance in combat athletes. We aimed to critically review clinical trial evidence on the impact of β-alanine supplementation on sports performance, perception, and anthropometric parameters, as well as circulating biochemical markers in combat athletes. This systematic review was conducted following the specific methodological guidelines of the Preferred Report Items for Systematic Reviews and Meta-Analyses guidelines (PRISMA), the PICOS question model, the Critical Review Form of McMaster, and the PEDro scale. Furthermore, the Cochrane risk-of-bias assessment tool was used. The search was carried out in the SCOPUS, Web of Science (WOS), and Medline (PubMed) databases for studies published from the beginning of the database until July 31, 2023. Of the 41 registers identified, only 7 met the established criteria and were included in this systematic review. Overall, performance parameters related to strength, power, total exercise work capacity, and combat-specific parameters were significantly improved (*p* < 0.05). Perception parameters increased non-significantly (*p* > 0.05). Regarding biochemical parameters, carnosine increased significantly (*p* < 0.05), pH decreased non-significantly (*p* > 0.05), and the results for blood bicarbonate and blood lactate were heterogeneous. Finally, there was a non-significant (*p* > 0.05) improvement in the anthropometric parameters of lean mass and fat mass. β-alanine supplementation appears to be safe and could be a suitable nutritional ergogenic aid for combat athletes.

## 1. Introduction

Combat sports can be classified into thre groups: grappling, hitting, and mixed. Grappling sports are characterized by holds, locks, and falls to the ground (e.g., judo, wrestling, and jiu-jitsu). Striking sports focus on the use of punches and kicks (e.g., kickboxing, boxing, Muay Thai, karate, taekwondo). Finally, mixed combat sports are those that have characteristics of both groups (e.g., hapkido, mixed martial arts [MMA]) [1]. These modalities of combat sports require the performance of explosive and high-intensity movements of both the upper and lower extremities [2]. The performance of combat sports occurs in short periods of time, from seconds to a few minutes, depending on the specific regulations of each one [3]. Overall, combat sports are sports of intermittent exertion because of the effort pattern and the “exercise-relative recovery” sequence [4]. Efforts in combat sports are characterized by intermittently demanding high work alternating short but intense explosive force and power actions that require good participation of anaerobic energy [5]. In this sense, high-intensity actions imply the intervention of anaerobic metabolism using the energy pathway of intramuscular adenosine triphosphate (ATP) and phosphocreatine (PCr) and/or short-term anaerobic glycolysis during exercise performance [1]. This metabolic situation causes high blood lactate levels after each combat. The accumulation of lactic acid can be important in a complete fight, but when several fights are carried out in a row, the acidosis caused by these can be detrimental to final performance [5]. Thus, combat sports practiced at a high level are long-term intermittent effort sports activities [4]. 

There is a great limitation of high-intensity maximum efforts because they can only be maintained for short periods of time [6] due to the appearance of short-term muscle fatigue, especially in the muscles involved in the exercise, which generates dysfunctions and discomfort that culminates in stopping the exercise [7]. Fatigue has metabolic consequences, such as a decrease in intramuscular PCr or an increase in lactate and a decrease in pH [7]. Muscle fatigue, then, is associated with, among other aspects, a rapid increase in the production of metabolic acids [8]. In the organism, there are immediate defense mechanisms; to avoid changes in the pH in response to changes in the acidity of body fluids, they are carried out by the buffer systems of the body, such as bicarbonate, phosphate, and hemoglobin, in addition to respiratory regulation and renal pH regulation [9]. The normal metabolism of the body continually produces acid radicals. In addition, this production increases during maximum-intensity exercises, causing situations of physiological stress, in which the buffer systems are not capable of restoring an electrolyte imbalance caused mainly by the production of lactic acid. Under these situations, athletes increase the risks of undergoing lactic acidosis, fatigue, and/or overtraining [7,8]. 

Therefore, it seems reasonable to implement nutritional ergogenic aids (NEAs) with a buffering capacity, helping to restore homeostatic balance and neutralize the rapid increase in the production of metabolic acids induced by high-intensity exercise [10]. NEAs have ergo-nutritional ingredients whose purpose is to help cover the specific nutritional requirements of combat sport practitioners, both for maintaining a good state of health and maximizing sports performance [11]. In this way, β-alanine is a non-essential amino acid synthesized in the liver. β-alanine does not have an ergogenic effect by itself, but it does as a precursor for the synthesis of carnosine (β-alanine and L-histidine) in human skeletal muscle [12]. Carnosine improves muscle contraction, increasing the sensitivity of myofibrillar calcium in fast fibers, and mediates 8–15% of the intramyocyte buffer capacity, reducing the limiting effect of performance related to acidosis [13]. β-alanine is a NEA with a degree of evidence A that significantly increases the concentrations of carnosine in the muscle, thus acting as an intracellular pH buffer [14,15]. In addition, it has been reported that exercise performance is improved, with more pronounced effects in activities lasting 1–4 min at doses of 4–6 g/day for at least 2–4 weeks, with a significant increase in carnosine from 20 to 30% and 40 to 60%, and after 10 weeks, an approximate increase of 80%. β-alanine has shown a moderately elevated ergogenic effect on the attenuation of neuromuscular fatigue [12,16]. Although it does not cause alterations in healthy populations at the recommended doses, a sensation of paresthesia in the extremities has been reported [17,18], as well as the appearance of itching due to L-alanine [18]. 

The consumption of NEAs has increased exponentially with prospects for the next decade, with an increase of between 10 and 15% [19]. Elite or recreational athletes, regardless of gender, consume NEAs equally [20]. Therefore, it is necessary to dispel doubts about the potential ergo-nutritional effect of β-alanine in combat sports, which obtained 26% of all medals at the Tokyo 2020 Olympic Games [21]. If we also add an increase in research interest in NEAs, it might be necessary to offer appropriate evidence-based advice by critically reviewing published randomized controlled trials (RCTs) on outcomes that are commonly investigated in sports nutrition science. We used the research question using the PICO model following the Evidence-Based Medicine (EBM) guidelines [22] as follows: P (population): “combat competition athletes who did not present chronic pathology”; I (intervention): “β-alanine supplementation”; C (comparison): “same conditions with placebo or control group”; O (outcomes): sports performance (strength, power, total exercise work capacity, vertical jump, and combat-specific parameters); perceptual parameters (perceptive exertion [CR-10 RPE scale], and better perceived exercise recovery [TQR scale 6–20]); anthropometric measures (lean mass, and fat mass); biochemical markers (serum carnosine, bicarbonate [HCO_3_]; pH and blood lactate [LAC]); and side effects (paresthesia). This systematic review was eligible for PROSPERO registration (#CRD42023426545) and was conducted and reported according to the PRISMA (Preferred Reporting Items for Systematic Reviews and Meta-analyses) guidelines [23]. These results were included as outcomes because these parameters are commonly investigated in sports science and nutrition studies to determine evidence for NEAs.

## 2. Materials and Methods

### 2.1. Search Methods

Our systematic review asked the following question: “Does β-alanine supplementation have positive impacts on sports performance, perception, and anthropometric parameters, and biochemical biomarkers for healthy combat athletes?” To answer this question, a structured search was carried out using the Scopus, Web of Science (WOS), and Medline (PubMed) database for studies published from the beginning of the database until 31 July 2023.

The search strategy contained terms related to β-alanine and free words for key concepts related that included the following: (“β-alanine OR Beta-alanine”) AND (“combat sports” or “judo” or “taekwondo” or “boxing” or “karate” or “wrestling”), AND (“supplementation” or “ergogenic aids”) AND (“combat sports” or “judo” or “taekwondo” or “boxing” or “karate” or “wrestling”) (Appendix A). In addition, we manually screened references from previous systematic reviews and meta-analyses and other sources, such as ResearchGate^®^ (https://www.researchgate.net/) (accessed on 24 June 2023), to find possible additional studies. Two reviewers (D.F.-L. and E.M.F.). independently assessed the full texts. In addition, a third reviewer (J.M.-A.) resolved the discrepancies. To identify potential studies not included in the databases, a network graph was made using the Connected Papers website (www.connectedpapers.com, accessed on 30 June 2023).

### 2.2. Elegibility Criteria

We based the collection of studies applying the following selection criteria: (i) healthy adult combat athletes; (ii) studies exclusively evaluating the use of β-alanine monotherapy supplementation in combat sports; (iii) comparing it with the control group, placebo group, or sham treatment (excluding comparisons with other supplements); (iv) studies with a methodological design that corresponds exclusively to a clinical trial; (v) studies that assessed sports performance, perceptual, and anthropometric parameters, as well as biochemical biomarkers, as outcomes; (vi) studies with clear information on the dose and duration of β-alanine supplementation; (vii) studies with a risk-of-bias score ≥ 4 according to the Cochrane Collaboration tool [24]; (viii) studies with a methodological quality score ≥ 13 according to the McMaster University Occupational Therapy Evidence-Based [25]; (ix) clinical trials or randomized clinical trials with a score ≥ 6 on the Physiotherapy Evidence Database (PEDro) scale [26]; and (x) studies published in Spanish or English. Those studies that did not meet the inclusion criteria that are described were eliminated. 

### 2.3. Methodological Quality and Risk-of-Bias Assessment

The methodological quality of the studies was evaluated by the McMaster tool [25] and PEDro scale [26]. Also, the Cochrane risk-of-bias tool was used [24]. 

### 2.4. Data Extraction

The following data were extracted from the selected studies: name of the first author; year of publication; country; study design; sample size; characteristics of the participants (gender and level of physical activity); intervention (daily amount of supplementation and timing of intake); and analyzed variables and results. This was carried out according to the CONSORT Statement rules [27]. To develop the data extraction, two components (D.F.-L. and E.M.F.) of the research team and another member (J.M.-A.) resolved the disagreements generated. 

## 3. Results

### 3.1. Study Selection

**Figure 1:**
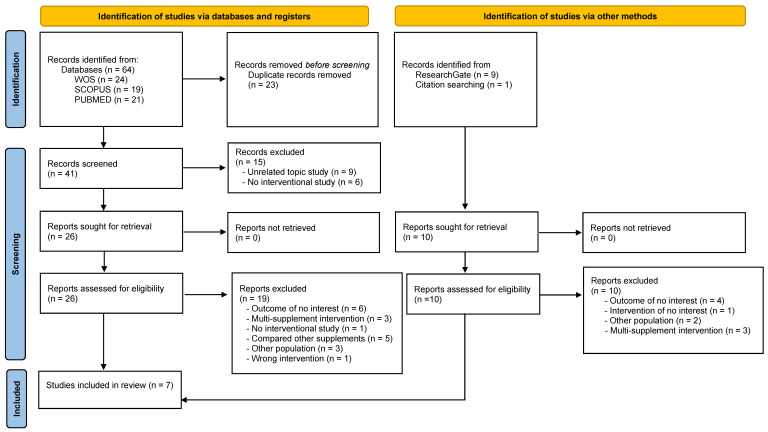
Flowchart representing the identification and selection processes of relevant studies according to the PRISMA 2020 declaration [23].

A total of 64 studies were identified; 31 studies were from three electronic databases, namely Web of Science, Scopus, and Medline (PubMed), and 10 studies were obtained from other sources, such as ResearchGate^®^. After excluding 23 duplicates, a total of 41 articles were evaluated. After title and abstract evaluation, 18 articles were considered potential registries. After reviewing the full text and assessing potential records from databases, registries, and other sources, seven studies [28,29,30,31,32,33,34] were included in the systematic review (Figure 1).

**Figure 2:**
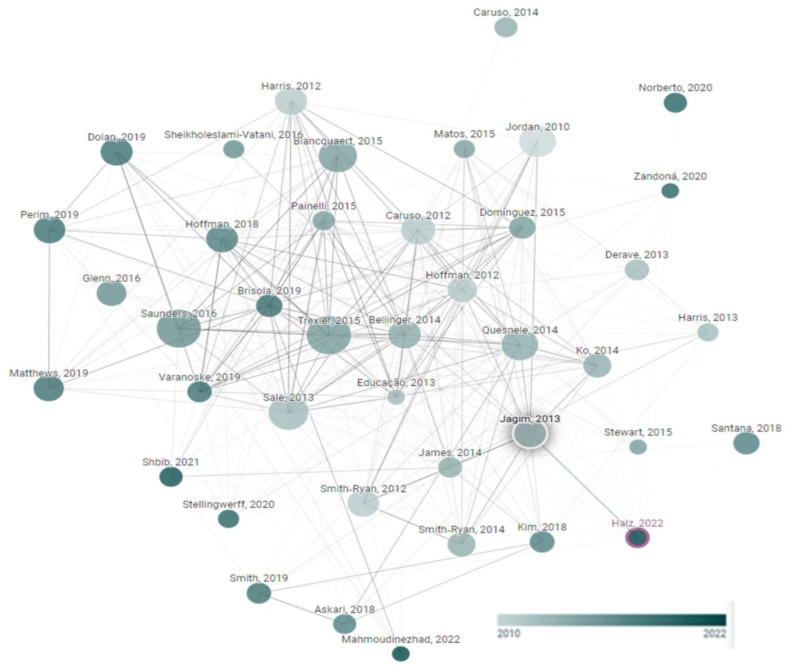
Network diagram of the β-alanine supplementation trials. This graph was developed within www.connectedpapers.com and accessed on 31 July 2023.

Figure 2 shows the node plot that originated from the research of Halz et al. [31]. Figure 2 was made to verify the studies included in this systematic review.

### 3.2. Quality Assessment

The evaluation of the methodological quality by the McMaster [25] tool yielded the following results: three studies [29,31,33] achieved a quality of “very good” and four studies [28,30,32,34] achieve a quality of “excellent”. Seven studies [28,29,30,31,32,33,34] reunited the minimum quality criteria (Table 1).

The methodological quality of the studies using the PEDro scale [26] was as follows: three studies [28,29,32] achieved ‘excellent’ quality and four studies [30,31,33,34] achieved ‘good’ quality (Table 2).

### 3.3. Risk-of-Bias Assessment

Two studies [30,34] had a score of “five points”, and five studies [28,29,31,32,33] had a score of “six points” (Table 3) in terms of risk of bias according to Cochrane Bias Methods Group [24].

### 3.4. Characteristics of the Participants and Interventions

The studies [28,29,30,31,32,33,34] included in our systematic review provided a total sample of 138 participants, 135 men [28,29,30,31,32,33,34] and 3 women [34]. In this way, 54 participants practiced boxing [28,30,33], 22 competed in wrestling [32], and 47 subjects were judokas [29,31,34]. The sporting levels of the participants were amateur athletes [28,30], competition athletes [29,31,32], and elite athletes [33,34]. The administration of β-alanine was orally via capsules in all studies [28,29,30,31,32,33,34] included in the systematic review. Doses ranged from 4 g/day [32] to 6.4 g/day [29] and 0.3 g/kg/day [≈20–28 g/day] [28]. According to Kern et al. [32], the dose was administered twice a day; in three studies [31,33,34], the doses were divided into three doses a day; in two studies [29,30], the doses were divided four times a day; and in one study [28], the dose was not specified. The timing of supplementation was not specified in two studies [28,30]; in three studies [29,31,34], it was to be taken with main meals; in one study [33], it was to be taken immediately after the main meals; and in the study conducted by Kern et al. [32], it was to be taken at breakfast and lunch. Finally, the duration ranged from four weeks [28,29,30,31] to ten weeks [33] (Table 4).

### 3.5. Outcome Assessment

The results of the registries selected in the systematic review are presented in Table 5.

#### 3.5.1. Sport Performance

The sports performance outcomes described in the seven selected studies [28,29,30,31,32,33,34] were strength, power, total work, heart rate, jump height, blood lactate concentration, and combat-specific parameters for each sport.

- Strength

Donovan et al. [30] evaluated the cumulative strength and the average blow strength. Both parameters, cumulative and blow strength, increased significantly (*p* < 0.05) compared to the control group (CG), as well as the group supplemented with β-alanine (BaG) and BaG, with respect to the baseline, before the BaG was supplemented. Also, the study conducted by Kim et al. [33] evaluated knee extension force and an increase without statistical significance (*p* > 0.05) was only observed in the left knee in the BaG compared to the CG. However, these authors have reported a significant increase (*p* < 0.05) in the BaG compared to pre-supplementation.

- Power

Kern et al. [32] evaluated power through two tests: the first consisted of running 300 yards (274.32 m) (anaerobic power) and the second consisted of hanging from a bar, keeping the arms at a 90° angle (muscular power). β-alanine-supplemented wrestlers significantly (*p* < 0.05) improved anaerobic and muscular power relative to the CG and the study baseline for BaG.

Peak power was evaluated in two studies [28,33]. Kim et al. [33] demonstrated a significant (*p* < 0.05) increase in lower body peak power in the BaG compared to the CG and a non-significant (*p* > 0.05) increase compared to the BaG prior to intervention. However, Alabsi et al. [28] did not observe any change in maximum power. The peak power drop [(peak power − minimum)/peak power × 100] was evaluated in two studies [28,33]. Therefore, lower values indicate better sports performance. In the study carried out by Alabsi et al. [28], the drop in power decreased in a non-significant (*p* > 0.05) way in the BaG compared to the CG; however, it did decrease significantly (*p* < 0.05) in comparison with the BaG before supplementation. Kim et al. [33] reported that it decreased significantly (*p* < 0.05) when comparing the BaG with the placebo group, and there was no change compared to the BaG at baseline.

Mean power was analyzed in two studies [28,31]. Alabsi et al. [28] did not find differences in mean power when comparing the BaG to the CG. However, these authors [28] reported a significant increase (*p* < 0.05) in mean power in the BaG after 10 weeks of supplementation with β-alanine. Halz et al. [22] did not observe changes in mean lower body power in the BaG with respect to the CG, but these authors [22] reported significant increases (*p* < 0.05) in mean upper body power when comparing the BaG to the non-supplemented group.

- Total exercise work capacity

Halz et al. [31] assessed the total exercise work capacity on the upper and lower extremities. In both the upper and lower extremities, total exercise work capacity was significantly increased (*p* < 0.05) in the BaG compared to the placebo group.

- Heart rate

Heart rate was evaluated in one study conducted by Donovan et al. [30] without observing changes while comparing both groups, namely the BaG and the CG.

- Vertical Jump

Vertical jump was studied in the clinical trial of Kim et al. [33]. It increased non-significantly (*p* > 0.05) when comparing the BaG to CG. However, in the BaG, vertical jump increased significantly (*p* < 0.05) between the beginning and the end of the study.

- Combat-specific parameters

The number of projections was evaluated in two studies [29,34]. In both studies [29,34], the number of projections increased, but only in the study conducted by de Andrade et al. [29] was a significant increase (*p* < 0.05) in the BaG with respect to the CG reported. The number of strokes was evaluated in the study conducted by Donovan et al. [30], being significantly higher (*p* < 0.05) in the BaG than in the placebo group. 

#### 3.5.2. Perception Parameters

Lopez-Grueso et al. [34] evaluated two perceptual parameters, namely perceived exertion (CR-10 RPE scale), and better perceived exercise recovery (TQR 6–20). Both parameters, CR-10 RPE and TQR 6–20, increased without statistical significance (*p* > 0.05) in the BaG compared to the CG. 

#### 3.5.3. Anthropometric Parameters

Lean mass (skeletal muscle, other types of muscle, and non-fat components) and fat mass (group of lipids or integral fats) were evaluated in the study conducted by Kern et al. [32] in wrestlers. Lean mass increased non-significantly (*p* > 0.05) and fat mass decreased non-significantly (*p* > 0.05) in the BaG compared to the CG and compared to the BaG vs. baseline.

#### 3.5.4. Biochemical Biomarkers

The circulating biochemical parameters evaluated were serum carnosine, HCO_3_, pH, and blood lactate [28,29,30,31,32,33,34].

- Serum Carnosine

Blood carnosine was measured in the study by Alabsi et al. [28], increasing significantly (*p* < 0.05) in the BaG compared to the CG.

- Bicarbonate (HCO3)

HCO_3_ was measured in two studies [29,31]. de Andrade et al. [29] observed a significant decrease (*p* < 0.05) in bicarbonate in the BaG with respect to its baseline. These authors [29] found no changes in bicarbonate concentration when comparing both groups, i.e., BaG vs. GC. However, in the study conducted by Halz et al. [31], HCO_3_ levels increased significantly (*p* < 0.05) in the BaG compared to the CG and with respect to baseline.

- pH

de Andrade et al. [29] showed that pH only decreased non-significantly (*p* > 0.05) in the BaG compared to pre-supplementation. No changes in pH were observed in the BaG vs. the CG.

- Blood Lactate

Blood lactate was measured in all selected studies [28,29,30,31,32,33,34] in this systematic review. Overall, the blood lactate concentration responses were heterogeneous when both groups were compared, namely the BaG and the CG. Four studies [28,29,33,34] did not observe changes; in two studies [30,31], a significant increase (*p* < 0.05) was observed; and Kern et al. [32] described a notable decrease (*p* > 0.05) in blood lactate concentration. 

With respect to blood lactate concentration measurements when evaluating the BaG with respect to its linear base, in five studies [28,29,30,31,33], it increased significantly (*p* < 0.05); in one study [34], no changes were observed; and only the study conducted by Kern et al. [32] in wrestlers observed a moderate decrease (*p* > 0.05) in blood lactate concentration.

#### 3.5.5. Adverse Effects

Two studies [29,34] reported cases of mild paresthesia.

## 4. Discussion

Our systematic review aimed to evaluate the effects of β-alanine supplementation on sports performance, anthropometric, and perception parameters, as well as biochemical markers, in healthy adults practicing combat sports. A total of seven studies [28,29,30,31,32,33,34] met the inclusion criteria, with 138 participants, 135 men [28,29,30,31,32,33,34], and 3 women [34]. In general, all the selected studies [28,29,30,31,32,33,34] showed significant improvements in sports performance in terms of strength, power, and total work capacity, better perception of recovery from physical exertion, and an increase in lean mass and decrease in fat mass in combat athletes after periods of β-alanine supplementation. On the other hand, β-alanine supplementation did not show conclusive evidence on the results related to certain circulating biochemical parameters and blood lactate concentration. Supplementation with β-alanine was shown to be safe since there were four dropouts [33,34] due to injury, not related to the supplementation, although mild paresthesia was manifested [29,34]. 

### 4.1. β-Alanine Supplementation

β-alanine supplementation was administered by oral capsules [28,29,30,31,32,33,34] and β-alanine is doping-free [35]. Doses ranged from 4 g/day [32] to 6.4 g/day [29] and 0.3 g/kg/day [≈20–28 g/day] [28]. Recently, Sport Integrity Australia [36] recommended that β-alanine supplementation should be started with a loading phase of 3.2 g per day for eight weeks, or 6.4 g per day for four weeks, followed by a maintenance β-alanine supplementation of 1.2 g per day. In this sense, Naderi et al. [18] reported that β-alanine supplementation of 1.2 g/day could maintain muscle carnosine in the range of 30% to 50% above pre-supplementation levels. It should be considered that intracellular carnosine levels are mainly determined by the availability of extracellular β-alanine [36]. Even more so, histidine could be supplemented to enhance intracellular carnosine stores [37]. 

The timing of β-alanine supplementation in the studies included in the review was with main meals [29,31,34], immediately after main meals [33], and with breakfast and lunch [32]. β-alanine supplementation during carbohydrate- and protein-rich meals markedly increased muscle carnosine content compared with β-alanine supplementation between meals. Perhaps insulin could induce β-alanine uptake by stimulating a greater carnosine load in muscle through the action of Na^+^/K^+^ pumps present in skeletal muscle myocytes [38].

Mild paresthesia in the extremities [29,34] was the only reported side effect of β-alanine supplementation. Paranesthesia is a consequence of an increase in the sensitivity of neuropathic pain-transmitting nociceptive neurons, which causes redness and an itching sensation on the skin [13,39]. Paranesthesia could be attenuated by fractionated lower doses (1.6 g per dose, in six–eight doses) or sustained release formulas [13] and consuming them with main meals to help improve absorption and better manage potential side effects [13,40]. 

### 4.2. Sports Performance

Increases in metabolic acids during intense physical activity is because muscle contraction substantially increases intracellular hydrogen ions and the extraordinary metabolic demands that are covered predominantly by anaerobic glycolysis, producing lactic acid [41]. Consequently, there is a decrease in the pH of the muscles that are exercised, which limits contractile function and muscle metabolism, significantly decreasing tolerance to exercise [42]. Faced with this situation, acid-base imbalance, the organism intrinsically possesses a capacity to fight against acidosis through the buffer or damping system [9]. However, high-intensity exercise exceeds this buffering capacity and, therefore, muscle fatigue is triggered, impairing the athlete’s sports performance [42]. 

Thus, β-alanine may improve performance by reducing acidity [43]. However, the results were conflicting, with some showing better performance in high-intensity exercise and others finding no difference [44,45]. One of the limiting factors in the efficacy of β-alanine in sports performance is acidosis. β-alanine could improve performance in physical actions that cause an extreme intramuscular acidotic environment [44]. However, the improvement in sports performance is limited when the exercise protocol does not produce severe muscle acidosis [46].

There was a significant (*p* < 0.05) performance improvement in strength [30], power [31,32,33], total exercise work capacity [31], and combat-specific parameters [29,30] with respect to the non-supplemented group. In the studies [29,30,31,32,33] included in our systematic review, the duration of the exercises or tests were from one to four minutes. Therefore, the main way of obtaining energy was anaerobic glycolytic, characterized by the production of LAC, creating an extreme acidotic environment [9]. Therefore, when comparing our results with other studies that investigate β-alanine supplementation in athletes, we must consider whether it is the main metabolic pathway for obtaining energy to play sports. Consistent with the results of our systematic review, the performance improvement associated with β-alanine supplementation also occurs in other sports with similar exercise times [47,48]. Ducker et al. [47] demonstrated a significant (*p* < 0.05) improvement in athletes who competed in 800 m races after four weeks of β-alanine supplementation with respect to the group. Also, in climbers after four weeks of supplementation with 4 g/day of β-alanine, performance improved during continuous climbs lasting one minute and repeated episodes of movements involving the upper extremities [48].

In this sense, Saunders et al. [45] described a significant (*p* < 0.05) improvement in sports performance in studies involving exercises between one minute to four minutes, with no improvement in exercises > one minute and with a slight improvement in exercises ranging from four minutes to 10 min. In exercises > one-minute duration, the main way of obtaining energy is the phosphagen system using phosphocreatine and, to a lesser extent, anaerobic glycolysis [9]. Therefore, in exercises lasting > one-minute, benefits in sports performance from the use of β-alanine are not evident [46,49,50] because this exercise duration is unlikely to be restricted by intracellular H^+^ increase [51]. 

On the other hand, regarding the physical exercises whose duration ranges from 4 to 10 min, both anaerobic glycolysis and the aerobic pathways are involved in its development [9]. Therefore, in these types of exercises in which anaerobic glycolysis is partly involved, slight benefits on sports performance are observed after β-alanine supplementation [52,53]. This could be justified because muscle carnosine would be the primary mechanism of the metabolic demand of exercise, as a pH buffer, which would only involve the anaerobic glycolysis pathway [52].

The use of β-alanine supplements does not appear to improve strength [44]. In athletes, increases in strength have been described after the use of creatine plus β-alanine supplement combinations [50], but not with β-alanine in monotherapy [49,50] or with other buffering supplements, such as HCO_3_ [54]. However, strength-related parameters improved from β-alanine supplementation compared to CG [30,33]. Donovan et al. [30] observed a significant (*p* < 0.05) improvement in cumulative force and mean punch force in boxers. Also, Kim et al. [33] observed a substantial increase in knee extension strength and vertical jump between the BaG and the CG. These findings may come as a surprise because strength performance is not limited by acidosis [18].

In three studies [31,32,33] included in the systematic review, significant (*p* < 0.05) improvements in power in the BaG vs. the CG were reported. Kim et al. [33] reported significant (*p* < 0.05) positive effects with a 6% improvement in maximal lower body power and a smaller 3.2% upper body power drop. Halz et al. [22] observed significant (*p* < 0.05) increases in mean power in the upper body. Additionally, Kern et al. [26] reported a significant (*p* < 0.05) improvement in anaerobic muscle power performance. Furthermore, in one study [28] included in the systematic review, significant improvements (*p* < 0.05) were described over a drop in maximum power and significant increases (*p* < 0.05) of 20% in mean power in the BaG compared to before intervention. These results are consistent with the study conducted by Van Thienen et al. [55], in which after eight weeks of oral supplementation with β-alanine, increases of 5% and 11.5% in mean power and maximum power were observed, respectively. However, other studies [49,50,56] did not show positive effects of β-alanine supplementation on power performance in upper arm flexion [49], squat exercises [50], or anaerobic muscle power during repeated sprint [56]. These differences could be because β-alanine improves performance in exercises that generate an extreme intramuscular acidotic environment [44]. But the probability of the effect of β-alanine decreases ostensibly with lower levels of acidosis [46]. PCr will be very present, and therefore, acidosis will not be the limiting factor in this type of exercise. The incomplete resynthesis of PCr has the greatest effect on fatigue and/or decreased performance than the accumulation of H^+^ [57]. 

Supplementation with β-alanine attenuates the appearance of muscular fatigue that would potentially improve total physical work capacity [44,45]. In judokas, significant (*p* < 0.05) increases in the total work in the upper and lower extremities [31] and significant (*p* < 0.05) increases in the total number of projections and projections per combat [29] in the BaG group with respect to the CG have been demonstrated. In addition, Lopez-Grueso et al. [34] described a remarkable tendency to increase in the BaG group, with respect to the CG, the number of total projections of judo. In boxers, the number of blows was evaluated in the study carried out by Donovan et al. [34], being significantly higher (*p* < 0.05) in the BaG group than in the placebo group. 

### 4.3. Anthropometric Parameters

An eight-week study, included in this systematic review, supplemented β-alanine (4 g/day), to collegiate wrestlers, and lean mass increased non-significantly (*p* > 0.05), while fat mass decreased non-significantly (*p* > 0.05) in the BaG compared to the CG and compared to the BaG vs. baseline [32]. These results are similar to those reported in a six-week study in athletic women, where the BaG (6 g β-alanine) saw an increase in lean mass, while the CG did not [58]. Similarly, a three-week study in 46 healthy men, supplemented with four 1.5 g doses of β-alanine (6 g/day), reported a significant increase in lean mass comparing the start of the study with the end of the study [59].

β-alanine may promote lean mass gains, but its mechanism is unknown. Perhaps the buffering capacity of β-alanine [43] makes it possible to support a greater volume of training, causing a greater stimulus; this leads to greater adaptations, and consequently, to an increase in muscle mass. Although, the anthropometric benefits could be a consequence of the exercise, since in the three studies [32,58,59], β-alanine supplementation was combined with an exercise regimen. This was reported previously in 2006 by Hoffman et al. [60]. These results are of interest for combat sports because the categories are separated by weight; the use of β-alanine can be a very interesting strategy to lower body fat and maintain or even increase lean mass [61]. 

### 4.4. Perception Parameters

β-alanine seems to reduce the perception of fatigue and delay voluntary exhaustion in women [62], older people (55–92 years) [62], and college athletes [63]. In the study included in our systematic review on judokas conducted by López-Grueso et al. [34], a discord between these parameters of subjective perception showing a non-significant (*p* > 0.05) increase in CR-10 RPE and a tendency (*p* > 0.05) to increase in the TQR 6–20 were shown. These discrepancies could be due to physiological differences between men and women, who also differ by at least one intensity of perceived exertion of exercise. In this sense [64], Hoffman et al. [63] also described discordance between fatigue as measured by subjective ratings and fatigue as measured by a Wingate anaerobic test. 

Furthermore, we should consider that perception is a biased parameter subject to subjectivity, feeling more tired when we lose than when we win. In this way, improvements in performance associated with β-alanine supplementation could determine a tendency to decrease the perception and sensation of fatigue [34].

### 4.5. Biochemical Biomarkers

In two studies [30,31], blood LAC had a significant increase (*p* < 0.05) in the BaG compared to the CG. Also, in five studies [28,29,30,31,33], blood LAC increased significantly (*p* < 0.05) in the BaG with respect to baseline. Blood LAC may be not the cause of H^+^ accumulation; the metabolic environment that causes a decrease in pH also increases lactate production, making LAC a good marker of conditions that induce metabolic acidosis [65], thus facilitating β-alanine action [44]. This increase in post-exercise LAC could be associated with β-alanine supplementation by counteracting the accumulation of H^+^, helping to maintain intramuscular pH during intense exercise [13]. Higher blood lactate levels could allow exercise to be carried out at a higher intensity for longer periods because it improves the buffering capacity [65]. This would allow the athlete to tolerate higher exercise loads, without the onset of fatigue at higher lactate levels [31]. Perhaps this could lead to a certain relationship between the improvements in performance parameters and the increase in LAC in the blood, as in the five studies [28,29,30,31,33] included in the systematic review.

Blood carnosine increased significantly (*p* < 0.05) in the BaG compared to the CG [28]. Increasing the intramuscular availability of β-alanine through supplementation is adequate to increase the endogenous synthesis of carnosine by carnosine synthetase [12]. Under normal physiological conditions, intramuscular β-alanine is below 40 μM (saturation point of carnosine synthetase), and therefore, the availability of β-alanine is the limiting factor of carnosine synthesis [66]. High concentrations of carnosine in the muscle are effective as an intracellular pH buffer [14,15]. In this way, de Andrade et al. [29] showed that pH only decreased non-significantly (*p* > 0.05) in the BaG compared to pre-supplementation. 

HCO_3_ levels increased significantly (*p* < 0.05) in the BaG compared to the CG and with respect to baseline [31]. The increases in HCO_3_ could be explained by the activation of this H^+^ buffer pathway [9]. The progress of exercise until fatigue produces a substantial amount of H^+^ in the blood [8] that is quickly captured by bicarbonate, forming carbonic acid (H_2_CO_3_), which quickly dissociates, giving rise to carbon dioxide (CO_2_) and water (H_2_O). This CO_2_ is driven to the lungs and expelled through breathing [9].

### 4.6. Adverse Effects

Two studies [29,34] included in our systematic review showed mild paresthesia. There are potential side effects associated with β-alanine, especially if a person takes it in large doses, although they are not severe. These may include skin rashes and paresthesia, a tingling sensation on the skin [17,18]. 

### 4.7. Limitations

The total sample of participants was small (*n* = 138), and only three female athletes were included. A small number of manuscripts were included because they met the inclusion criteria. In the seven records included, there is great variability in the β-alanine supplementation regimes, the sports modality, and the sports level of the athletes. In addition, the results of sports performance, perceptual, and anthropometric parameters, as well as biochemical markers were heterogeneous, which prevented the development of a meta-analysis. Also, the high risk of bias that could undermine confidence in the results should be considered because the studies included in this systematic review could overestimate or underestimate the true effect of β-alanine supplementation. For all the above, we recommend interpreting the results with caution.

### 4.8. Strengths

The systematic review was carried out following the PRISMA rules [23], and the search was conducted in three databases, namely PubMed, SCOPUS and WOS, and ResearchGate^®^. Two methodological quality tools were used, namely McMaster [25] and PEDro [26]. We also used the Cochrane risk-of-bias assessment instrument [24]; in addition, this review was recorded in PROSPERO (#CRD42023426545). 

## 5. Conclusions and Perspectives

β-alanine supplementation in a dose range of 4 g/day to 6 g/day for at least four weeks can improve athletic performance for high-intensity exercises lasting between 60 s and 240 s, which intramuscularly induce an extremely acidic environment. Taken together, the results described in our systematic review showed that β-alanine supplementation is safe with potential effects on performance in strength, power, and total exercise work capacity, as well as combat-specific parameters in combat athletes. Also, supplementation with β-alanine improves lean mass, decreases fat mass, and improves the feeling of recovery after a fight. These benefits were associated with the availability of β-alanine and carnosine, which is the product that forms β-alanine, to buffer H^+^ and with some antioxidant capacity. Therefore, β-alanine could be a suitable NEA for combat athletes seeking to improve their sports performance and anthropometric parameters, but more evidence is needed to confirm these findings. Considering the described results, supplementation with β-alanine could be beneficial in sports with the physiological characteristics simulating combat sports, such as high-intensity intermittent exercises and high-intensity exercises of more than one minute and less than four minutes and when fatigue is established as CrossFit, artistic and rhythmic gymnastics, middle-distance running in athletics, swimming, and rowing.

## Figures and Tables

**Table 1:** Results of the methodological quality assessment of included studies—McMaster Critical Review Form for Quantitative Studies [25].

Study, Year	Item																Total	%	Quality Score
	1	2	3	4	5	6	7	8	9	10	11	12	13	14	15	16			
Alabsi et al., 2022 [28]	1	1	1	1	1	0	1	1	1	1	1	1	1	1	1	0	15	93.8	E
De Andrade et al., 2017 [29]	1	1	1	1	1	0	1	1	0	1	1	1	1	1	1	0	14	87.5	VG
Donovan et al., 2012 [30]	1	1	1	1	1	1	0	1	1	1	1	1	1	1	1	1	15	93.8	E
Halz et al., 2022 [31]	1	1	1	1	1	0	1	1	1	1	1	1	1	1	1	0	15	93.8	E
Kern et al., 2011 [32]	1	1	1	1	1	0	1	1	1	1	1	1	1	1	1	0	14	87.5	VG
Kim et al., 2018 [33]	1	1	1	1	1	1	0	0	1	1	1	1	0	1	1	1	13	81.3	VG
López-Grueso et al., 2014 [34]	1	1	1	1	1	0	1	1	1	1	1	1	1	1	1	0	15	93.8	E

Abbreviations: 0 = not fulfilled criterion; 1 = fulfilled criterion; E = excellent; VG = very good; G = good; F = fair. Item 1: study purpose; item 2: literature review; item 3: study design; item: 4 blinding; item 5: sample description; item 6: sample size; item 7: ethics and consent; item 8: validity of outcomes; item 9: reliability of outcomes; item 10: intervention description; item 11: statistical significance; item 12: statistical analysis; item 13: clinical importance; item 14: conclusions; item 15: clinical implications; item 16: study limitations.

**Table 2:** Evaluation of methodological quality according to PEDro scale [26].

Study, Year	Items											Total	%	Quality Score
	1	2	3	4	5	6	7	8	9	10	11			
Alabsi et al., 2022 [28]	1	1	0	1	1	1	0	1	1	1	1	10	90.9	E
De Andrade et al., 2017 [29]	1	1	0	1	1	1	0	1	1	1	1	10	90.9	E
Donovan et al., 2012 [30]	1	0	0	1	1	0	0	1	1	1	1	7	63.64	G
Halz et al., 2022 [31]	1	0	0	1	1	1	0	1	1	1	1	8	72.7	G
Kern et al., 2011 [32]	1	1	0	1	1	1	0	1	1	1	1	9	81.82	E
Kim et al., 2018 [33]	1	0	0	1	1	1	0	1	1	1	1	8	72.7	G
López-Grueso et al., 2014 [34]	1	0	0	1	1	0	0	0	1	1	1	6	54.5	G

Abbreviations: 0 = not fulfilled criterion; 1 = fulfilled criterion; E = excellent; VG = very good; G =good; F = fair. Item 1: eligibility criteria were specified; item 2: subjects were randomly allocated to groups; item 3: allocation was concealed; item 4: the groups were similar at baseline regarding the most important prognostic indicators; item 5: there was blinding of all subjects; item 6: there was blinding of all therapists who administered the therapy; item 7: there was blinding of all assessors who measured at least one key outcome; item 8: measures of at least one key outcome were obtained from more than 85% of the subjects initially allocated to groups; item 9: all subjects for whom outcome measures were available received the treatment or control condition as allocated or, where this was not the case, data for at least one key outcome were analyzed by “intention to treat”; item 10: the results of between-group statistical comparisons are reported for at least one key outcome; item 11: the study provides both point measures and measures of variability for at least one key outcome.

**Table 3:** Results of the risk-of-bias assessment of included studies—Cochrane Bias Methods Group [24].

Study, Year	Items								Total
	1	2	3	4	5	6	7	8	
Alabsi et al., 2022 [28]	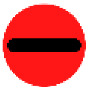	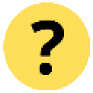	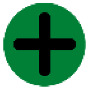	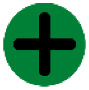	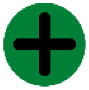	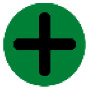	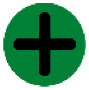	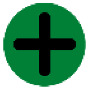	6
De Andrade et al., 2017 [29]	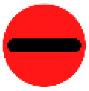	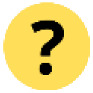	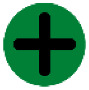	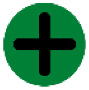	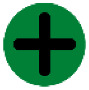	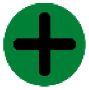	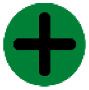	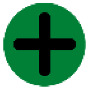	6
Donovan et al., 2012 [30]	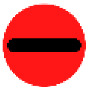	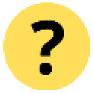	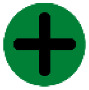	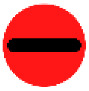	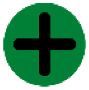	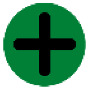	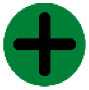	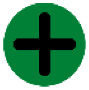	5
Halz et al., 2022 [31]	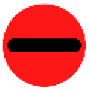	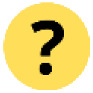	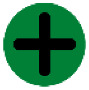	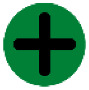	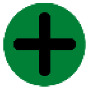	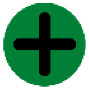	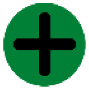	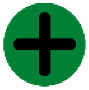	6
Kern et al., 2011 [32]	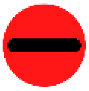	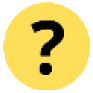	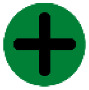	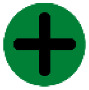	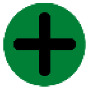	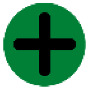	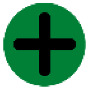	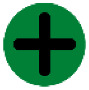	6
Kim et al., 2018 [33]	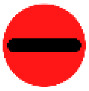	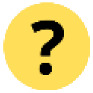	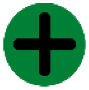	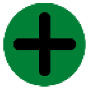	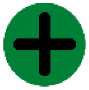	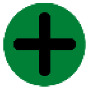	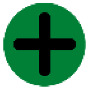	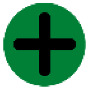	6
López-Grueso et al., 2014 [34]	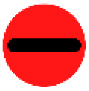	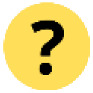	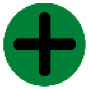	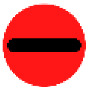	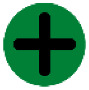	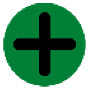	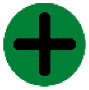	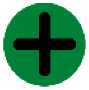	5

Abbreviations = 1: generation of sequences; 2: allocation concealment; 3: personal blinding; 4: blinding of assessor; 5: incomplete follow-up; 6: data report; 7: publication bias; 8: observer bias. The rating for each item includes the answer to one question, where “+” indicates bias, “f” indicates low risk, “−” indicates high risk of bias, and “?” indicates lack of information or uncertainty about the potential for bias; the higher the score, the greater the risk of bias.

**Table 4:** Characteristics of athletes and β-alanine supplementation practice of the included records.

Characteristics	Types	Study
Level of participants	Amateur athletes	[28,30]
	Competition athletes	[29,31,32]
	Elite athletes	[33,34]
Pharmaceutical form	Oral supplementation by capsules	[28,29,30,31,32,33,34]
Dosages used	0.3 g/kg/ day	[28]
	4 g/day	[32]
	4.9 g/day or 5.4 g/day	[33]
	6 g/day	[30,34]
	6.4 g/day	[29]
	4 g/day/2 weeks + 6 g/day/2 weeks	[31]
Divided dose in the day	2 times a day	[32]
	3 times a day	[31,33,34]
	4 times a day	[29,30]
	Unspecified	[28]
Dose schedule	With the main meals	[29,31,34]
	Immediately after main meals	[33]
	Breakfast and lunch	[32]
	Unspecified	[28,30]
Duration (weeks)	4	[28,29,30,31,34]
	8	[32]
	10	[33]

Abbreviations: g = grams; kg = kilograms.

**Table 5:** Records included in the systematic review of the effect of β-alanine supplementation on sports performance, perceptual, and anthropometric parameters, as well as biochemical markers in combat sports.

First Author, Year of Publication, and Country	Study Design	Participants (Baseline Sample Size, Age, Sex, Withdrawals, and Final Group Sample Size)	Intervention	Outcomes	Results
Alabsi et al. [28], 2022, Iran	Randomized, double-blind crossover, placebo-controlled trial	18 ♂ well-trained Korean boxers BA = *n* = 9 Age (mean ± SD) 24.44 ± 5.76 years Height (mean ± SD) 78.66 ± 3.31 cm Body mass (mean ± SD) 80.95 ± 13.74 kg BMI (mean ± SD) 22.88 ± 3.33 kg/m^2^ Fat mass (mean ± SD) 23.01 ± 3.20%. PLA: *n* = 9 Age (mean ± SD) 22.00 ± 4.69 years Height (mean ± SD) 173.77 ± 4.26 cm Body mass (mean ± SD) 69.13 ± 10.75 kg BMI (mean ± SD) 25.3 0 ± 3.72 kg/m^2^ Fat mass (mean ± SD) 15.14 ± 6.99%. Study withdrawals: 0	0.3 g/kg of BA or PLA (maltodextrin) Encapsulated in 800 mg capsules Supplementation time: 4 weeks	MaxP AP MPD CAR in blood LAC in blood	*BA* vs. *PLA* ↔ MaxP ↔ AP ↓ MPD ↑* CAR in blood ↔ LAC in blood *BA* vs. *Pre-Supple* ↔ MaxP ↑* AP ↓* MPD ↑* CAR in blood ↑* LAC in blood
de Andrade et al. [29], 2017, Brazil	Randomized, double-blind crossover, placebo-controlled trial	23 ♂ judo athletes BA: *n* = 12 Age (mean ± SD) 17 ± 2 years Body mass (mean ± SD) 74.2 0 ± 11.60 kg Experience (mean ± SD) 9 ± 3 years PLA: *n* = 11 Age (mean ± SD) 19 ± 3 years Body mass (mean ± SD) 71.5 0 ± 10.70 kg Experience (mean ± SD) 11 ± 4 years Study withdrawals: 0	6.4 g/day of BA or PLA (dextrose) Encapsulated in 800 mg capsules (4 times daily) Supplementation time: 4 weeks	P x C TP Blood pH LAC in blood HCO_3_ in blood	*BA* vs. *PLA* ↑* P x C ↑* TP ↔ Blood pH ↔ LAC in blood ↔ HCO_3_ in blood *BA* vs. *Pre-Supple* ↑* P x C ↑* TP ↓ Blood pH ↑* LAC in blood ↓* HCO_3_ in blood
Donovan et al. [30], 2012, United Kingdom	Randomized, controlled, single-blind trial	16 ♂ boxing competitors BA: *n* = 8, PLA: *n* = 8 Age (mean ± SD) 25 ± 4 years Height (mean ± SD) 1.74 ± 0.07 m Body mass (mean ± SD) 78.4 0 ± 7.60 kg 25 ± 4 years, 78.4 0 ± 7.60 kg, 1.74 ± 0.07 m Study withdrawals: 0	6 g/day of encapsulated BA or PLA (maltodextrin) divided into 4 doses per day (1.5 g) Supplementation time: 4 weeks	HR LAC in blood MedF TH AS	*BA* vs. *PLA* ↔ HR ↑* LAC in blood ↑* MedF ↑* TH ↑* AS *BA* vs. *Pre-Supple* ↔ HR ↑* LAC in blood ↑* MedF ↑* TH ↑* AS
Halz et al. [31], 2022, Poland	Randomized, double-blind crossover, placebo-controlled trial	16 ♂ elite judo athletes BA: *n* = 8 Age (mean ± SD) 20.7 0 ± 3.20 years Height (mean ± SD) 177.2 0 ± 2.60 cm Body mass (mean ± SD) 81.5 0 ± 3.90 kg VO_2max_ (mean ± SD) 54.5 0 ± 3.80 mL/kg/min Fat mass (mean ± SD) 10.90 ± 2.60% PLA: *n*= 8 Age (mean ± SD) 22.1 0 ± 2.80 years Height (mean ± SD) 178.30 ± 4.90 cm Body mass (mean ± SD) 78.40 ± 5.10 kg VO_2max_ (mean ± SD) 52.60 ± 4.90 mL/kg/min Fat mass (mean ± SD) 9.80 ± 3.20% Study withdrawals: 0	4 g/day of BA or PLA for 2 weeks divided into 3 intakes 6 g/day of BA or PLA for 2 weeks divided into 3 intakes Supplementation time: 4 weeks	TLW TUW AVP lower AVP higher LAC in blood HCO_3_ in blood	*BA* vs. *PLA* ↑* TLW ↑* TUW ↔ AVP lower ↑* AVP superior ↑* LAC in blood ↑* HCO_3_ in blood *BA* vs. *Pre-Supple* ↑* TLW ↑* TUW ↔ AVP lower ↑* AVP superior ↑* LAC in blood ↑* HCO_3_ in blood
Kern et al. [32], 2011, USA	Randomized, double-blind crossover, placebo-controlled trial	37 ♂ wrestling and football competitors W BA: *n* = 10 Age (mean ± SD) 20.10 ± 2.06 years Height (mean ± SD) 174.0 0 ± 8.07 cm Body mass (mean ± SD) 73.8 0 ± 15.64 lbs W PLA: *n* = 12 Age (mean ± SD) 19.8 0 ± 1.83 years Height (mean ± SD) 174.80 ± 6.55 cm Body mass (mean ± SD) 77.60 ± 13.84 lbs Body mass (mean ± SD) Study withdrawals: 0	Dosage: 4 g/day of BA or PLA (dextrose) Encapsulated divided into 2 doses per day Supplementation time: 8 weeks	AP LAC LM FM	W BA vs. W PLA ↑* AP ↓ LAC ↑ LM ↓ FM W BA vs. W Pre-Supple ↑* AP ↓ LAC ↑ LM ↓ FM
Kim et al. [33], 2018, Korea	Double-blind crossover study	20 ♂ Korean boxing athletes BA: *n* = 10 Age (mean ± SD) 23.00 ± 1.82 years Height (mean ± SD) 180.41 ± 7.42 cm Body mass (mean ± SD) 77.25 ± 20.64 kg Fat mass (mean ± SD) 12.30 ± 7.89% BMI (mean ± SD) 23.6 ± 5.51 kg/m^2^ Experience (mean ± SD) 7.27 ± 0.95 years PLA: *n* = 10 Age (mean ± SD) 22.2 0 ± 2.21 years Height (mean ± SD) 178.59 ± 6.33 cm Body mass (mean ± SD) 75.31 ± 19.21 kg Fat mass (mean ± SD) 13.87 ± 6.44% BMI (mean ± SD) 24.03 ± 4.49 kg/m^2^ Experience (mean ± SD) 7.41 ± 0.73 years Study withdrawals: 1 (injury) BA *n* = 9	4.9 g/day of BA or PLA in capsules for 49–69 kg. 5.4 g/day of BA or PLA in capsules for 75–91 kg. In 3/ times a day (18–30 mg/kg/meal) Supplementation time: 10 weeks	MaxP lower MPD upper EF left knee VJ LAC in blood	BA vs. PLA ↑* MaxP upper ↓* MPD upper ↑ EF left knee ↑ VJ ↔ LAC in blood BA vs. Pre-Supple ↑ MaxP lower ↔ MPD upper ↑* EF left knee ↑* VJ ↑* LAC in blood
López-Grueso et al. [34], 2014, Spain	Quasi-experimental, single-blind trial	8 judokas of the Spanish judo team BA: *n* = 4 (3 ♂, 1 ♀) Age (mean ± SD) 23.50 ± 0.70 years Height (mean ± SD) 1.60 ± 0.04 cm Body mass (mean ± SD) 61.4 0 ± 1.40 kg PLA: *n* = 4 (2 ♂, 2 ♀) Age (mean ± SD) 25.0 0 ± 1.00 years Height (mean ± SD) 1.70 ± 0.04 cm Body mass (mean ± SD) 66.30 ± 9.90 kg Study withdrawals: 3 (injury) BA *n* = 2 (1 ♂, 1 ♀) PLA *n* = 3 (1 ♂, 2 ♀)	6 g/day of BA or PLA (maltodextrin) Encapsulated divided into 3 doses per day Supplementation time: 35 days	TP P PE PBR LAC in blood	BA vs. PLA ↑ TP ↑ P ↑ PE ↑ PBR ↔ LAC in blood BA vs. Pre-Supple ↑ TP ↑ P ↑ PE ↑ PBR ↔ LAC in blood

Abbreviations = AP: anaerobic power; AS: accumulated strength; AVP: average power; BA: beta-alanine; BMI: body mass index; CAR: carnosine; EF: extension force; HCO_3_: bicarbonate; FM: fat mass; HR: heart rate; Kg: kilos; LAC: lactate; LM: lean mass; MaxP: maximum power; MedF: medium strength; MPD: maximum power drop; PBR: perception of better recovery; P: performance; PE: perceived exertion; PLA: placebo; Pre-supple: compared to the same group supplemented with BA before being supplemented; P x C: projections per combat; TH: total hits; TP: total projections; TLW: total lower extremities work; TUW: upper extremities total work; VJ: vertical jump height (Sargent test); W: wrestling. ↑ no significant increase; ↓: no significant decrease; ↔: no significant change. ↑*: significant increase; ↓*: significant decrease; *: Indicates significant values (*p* < 0.05); ♂: males; ♀: females.

## Appendix A

The search sequences carried out in the Scopus, Web of Science and PubMed databases were as follows:

- Scopus

(TITLE-ABS-KEY (beta AND alanine) AND (TITLE-ABS-KEY (combat AND sport), (TITLE-ABS-KEY (beta AND alanine) AND (TITLE-ABS-KEY (judo), (TITLE-ABS-KEY (beta AND alanine) AND (TITLE-ABS-KEY (karate), (TITLE-ABS-KEY (beta AND alanine) AND (TITLE-ABS-KEY (taekwondo), (TITLE-ABS-KEY (beta AND alanine) AND (TITLE-ABS-KEY (boxing), (TITLE-ABS-KEY (beta AND alanine) AND (TITLE-ABS-KEY (boxer), (TITLE-ABS-KEY (beta AND alanine) AND (TITLE-ABS-KEY (wrestling), (TITLE-ABS-KEY (b AND alanine) AND (TITLE-ABS-KEY (combat AND sport), (TITLE-ABS-KEY (b AND alanine) AND (TITLE-ABS-KEY (judo), (TITLE-ABS-KEY (b AND alanine) AND (TITLE-ABS-KEY (karate), (TITLE-ABS-KEY (b AND alanine) AND (TITLE-ABS-KEY (taekwondo), (TITLE-ABS-KEY (b AND alanine) AND (TITLE-ABS-KEY (boxing), (TITLE-ABS-KEY (b AND alanine) AND (TITLE-ABS-KEY (boxer), (TITLE-ABS-KEY (b AND alanine) AND (TITLE-ABS-KEY (wrestling), (TITLE-ABS-KEY (supplementation AND alanine) AND (TITLE-ABS-KEY (combat AND sport), (TITLE-ABS-KEY (supplementation AND alanine) AND (TITLE-ABS-KEY (judo), (TITLE-ABS-KEY (supplementation AND alanine) AND (TITLE-ABS-KEY (karate), (TITLE-ABS-KEY (supplementation AND alanine) AND (TITLE-ABS-KEY (taekwondo), (TITLE-ABS-KEY (supplementation AND alanine) AND (TITLE-ABS-KEY (boxing), (TITLE-ABS-KEY (supplementation AND alanine) AND (TITLE-ABS-KEY (boxer), (TITLE-ABS-KEY (supplementation AND alanine) AND (TITLE-ABS-KEY (wrestling), (TITLE-ABS-KEY (ergogenic aid AND alanine) AND (TITLE-ABS-KEY (combat AND sport), (TITLE-ABS-KEY (ergogenic aid AND alanine) AND (TITLE-ABS-KEY (judo), (TITLE-ABS-KEY (ergogenic aid AND alanine) AND (TITLE-ABS-KEY (karate), (TITLE-ABS-KEY (ergogenic aid AND alanine) AND (TITLE-ABS-KEY (taekwondo), (TITLE-ABS-KEY (ergogenic aid AND alanine) AND (TITLE-ABS-KEY (boxing), (TITLE-ABS-KEY (ergogenic aid AND alanine) AND (TITLE-ABS-KEY (boxer), (TITLE-ABS-KEY (ergogenic aid AND alanine) AND (TITLE-ABS-KEY (wrestling).

- Web of Science

(ALL=(beta alanine)) AND ALL=(combat sport), (ALL=(beta alanine)) AND ALL=(boxing), (ALL=(beta alanine)) AND ALL=(boxer), (ALL=(beta alanine)) AND ALL=(judo), (ALL=(beta alanine)) AND ALL=(taekwondo), (ALL=(beta alanine)) AND ALL=(karate), (ALL=(supplementation)) AND ALL=(combat sport), (ALL=(supplementation)) AND ALL=(judo), (ALL=(supplementation)) AND ALL=(taekwondo), (ALL=(supplementation)) AND ALL=(wrestling), (ALL=(supplementation)) AND ALL=(boxing), (ALL=(supplementation)) AND ALL=(boxer), (ALL=(supplementation)) AND ALL=(karate), (ALL=(ergogenic aid)) AND ALL=(combat sport), (ALL=(ergogenic aid)) AND ALL=(judo), (ALL=(ergogenic aid)) AND ALL=(boxing), (ALL=(ergogenic aid)) AND ALL=(boxer), (ALL=(ergogenic aid)) AND ALL=(taekwondo)

- Pubmed

(beta alanine) AND (combat sport), (beta alanine) AND (judo), (beta alanine) AND (karate), (beta alanine) AND (taekwondo), (beta alanine) AND (boxing), (beta alanine) AND (boxer), (beta alanine) AND (wrestling), (b alanine) AND (combat sport), (b alanine) AND (judo), (b alanine) AND (karate), (b alanine) AND (taekwondo), (b alanine) AND (boxing), (b alanine) AND (boxer), (b alanine) AND (wrestling), (supplementation) AND (combat sport), (supplementation) AND (judo), (supplementation) AND (karate), (supplementation) AND (taekwondo), (supplementation) AND (boxing), (supplementation) AND (boxer), (supplementation) AND (wrestling), (ergogenic aid) AND (combat sport), (ergogenic aid) AND (judo), (ergogenic aid) AND (karate), (ergogenic aid) AND (taekwondo), (ergogenic aid) AND (boxing), (ergogenic aid) AND (boxer), (ergogenic aid) AND (wrestling).
